# Eliminating malaria in our time

**DOI:** 10.1371/journal.pgph.0003086

**Published:** 2024-04-17

**Authors:** Caroline A. Lynch, Karma Lhazeen, Manash Shrestha, Varunika Ruwanpura, Neena Valecha, Kamala Thriemer

**Affiliations:** 1 Medicines for Malaria Venture (MMV), Geneva, Switzerland; 2 Asia Pacific Malaria Elimination Network (APMEN) Vivax Working Group, Singapore, Singapore; 3 Department of Public Health, Ministry of Health, Thimphu, Bhutan; 4 Global and Tropical Health Division, Menzies School of Health Research and Charles Darwin University, Darwin, Australia; Ifakara Health Institute, UNITED REPUBLIC OF TANZANIA

We could be on the cusp of history—eliminating malaria in our lifetime. But to meet the World Health Organisation’s (WHO) Global Technical Strategy targets and stay on course for elimination, at least 30 more countries need to achieve their malaria elimination goals by 2030 [1]. To be certified malaria-free by WHO in 2030, countries must have no malaria transmission for at least three years prior to certification. This means they have four years.

Plasmodium falciparum and P. vivax are the most prevalent malaria species. Vivax is widespread across the Asia-Pacific, South America and the Horn of Africa. Its elimination is particularly challenging because of key differences in parasite biology, especially the dormant liver-stage causing relapse. Radical cure with 8-aminoquinolines (8-AQ), either primaquine or tafenoquine that treat dormant liver stages, is essential for elimination. Effective radical cure is limited by safety concerns for patients with an enzyme deficiency (G6PD) at risk of hemolysis from 8-AQ treatment; suboptimal dosing regimens [2, 3]; and a relatively long primaquine treatment course with low patient adherence. New tools are becoming available including shorter, higher dose primaquine [4], single-dose tafenoquine [5], and near-patient G6PD tests that will provide improved safety of 8-AQ use [6]. These may offer more effective treatments and some, such as tafenoquine and G6PD tests, are being introduced into routine care in several malaria endemic countries [7–9].

The processes required to move from evidence generation to patient access take time. National malaria treatment guideline changes can take between 1.5 and 13.5 years including global recommendations (1–7 years) [10], national regulatory approvals (5 months—3.5 years) and national policy change (3 months—3 years) [11]. The accumulation of processes at all levels means that often patients do not have timely access to new, innovative tools.

To address the complexity and related timeline issues involved in introducing new tools, in 2018, the WHO began to pilot a streamlined process for drug approvals that involved horizon-scanning for new medicines approved by stringent regulatory authorities (SRAs) and simultaneous, rather than sequential, prequalification and recommendation development for the medicines [10]. However, while tafenoquine received two SRA approvals in 2019 [12, 13], and was a pilot drug in the new WHO system, the stipulation in the tafenoquine label that a quantitative G6PD test is required ahead of its use, meant that the prequalification and recommendation of such a test was also required. The best current option for that test is a quantitative G6PD test (STANDARDTM) approved by the Australian Therapeutic and Goods Agency in 2021. The test was submitted to the WHO prequalification in Oct 2021. According to the current process, only after prequalification of the test will the WHO be able to issue their first global tafenoquine policy recommendation. National changes to antimalarial policy often follow global WHO recommendations. However, both national regulatory approval of the drug and revision of national policy are required, meaning that- new tools for vivax malaria may only be included into national policies by 2030 [10, 11].

## How can the global malaria community work together to accelerate these timelines?

Greater country ownership. Global-level evidence reviews could be limited to efficacy and safety with responsibility for assessment of feasibility of adoption and implementation formally transferred to country-level. This would give countries the space to further expand country expertise, increase National Malaria Programs’ (NMP) decision-making space and ownership over which options are best for their contexts [14], in alignment with the WHO’s and key donor partners’ principle of country ownership [1].

Accepting risk. Change and innovation bring risk and vulnerability. It is imperative that new tools and approaches are safe for patients, but we also need to recognise the risk of delaying access to the intervention. Balancing these two critical considerations is key. Proactive approaches to managing risk that also facilitate earlier access to new interventions are urgently needed.

Process and system innovations. New tools are most useful if we can get them into the hands of patients in a timely way–current processes do not allow for this. However, regulatory reliance models (Where a regulatory authority in one jurisdiction gives significant weight to work performed by another regulator or other trusted institution in reaching its own decision), joint dossier assessments (beyond proof of concept), streamlined policy change such as that being piloted by WHO, and higher-quality training processes are approaches that could shorten the timeframe for introducing new tools.

Planning and readiness for delivery. NMPs need to know when to expect global recommendations to plan for their national policy change process so that they can prepare systems in parallel to global processes. This would allow for potential challenges at implementation stage to be addressed ahead of time.

Proactive preparation of the health systems for new tools is not typically part of the daily routine for NMPs that are often focused on more immediate implementation. Given the time it takes to move from regulatory approvals to recommendations at global and national levels, more emphasis on readiness is key. Identifying specific components of the health system that require strengthening to enable effective use of new tools could greatly accelerate their uptake and reduce effectiveness decay [15].

With the threat of artemisinin resistance emerging in some African countries and drastic climate changes impacting health systems, we may have no other chance to eliminate malaria than now or risk a resurgence and step back to the situation of the early 2000s. Integrated planning of the path to patients, including parallel, expedited regulatory, prequalification and policy processes is key.

There is hope − the COVID pandemic accelerated new technologies now being used or developed for malaria prevention and treatment–long-acting injectables, malaria vaccines and monoclonal antibodies—and the vector control pipeline looks promising. Advances in molecular surveillance enable us to better detect changes in the parasite earlier. We could accelerate and sustain malaria elimination by changing the global health system, strengthening country ownership, embracing risk, speeding up implementation of process innovations, and improving readiness for new tools.
